# When Began We?

**Published:** 1862-03

**Authors:** 


**WHEN BEGAN WE?**—We end never! for the soul is immortal, and can not die. When the soul's existence commences is as yet a conjecture. Nor can we tell when the immaterial first takes up its dwelling with the material; when the soul enters the body. But this we do know, that, at a point when the man that is to be is so minute as to require the microscope to determine whether it exists or not, the first faint outlines of the new being are defined to be a nervous system. The very first step cognizable to us, which nature takes to make a man a living soul, is to prepare the machinery, so to speak, through which that soul is to manifest itself. It is the nervous system which first begins to live, and to appropriate to itself those materials of growth which eventually become the human body and make a man. Nothing can be clearer than that the nervous system of the new being is connected with and is dependent on that of the parent, and that the hues, the impressions of the young, depend on the character of those of the parent. If, at this time, the parent is in perfect health, and so remains, it is fair to presume that the child will be born in perfect health, body and mind. These statements make the strongest possible appeal to all who may become mothers, to make it their constant study, their steady aim and effort, to secure a healthful condition of the body and a state of mind which shall be uniformly all that the mother desires the child to possess—piety, integrity, dignity, and an elevation of soul, which proves relationship to the Infinite. If the mother that is to be, wishes her child to possess vigorous manly health, she must cultivate the strictest personal cleanliness, extending to the most minute item pertaining to the human body; she must eat with regularity, not oftener than thrice a day; she must keep her feet, by all possible means, always dry and warm; her sleep must be early, and of the greatest abundance that nature can possibly take, out of daylight; one half of each day should be spent in open air activities; and nearly all the time of in-doors should be employed in cheerful, interesting, active work, constantly diversified, so as not to overtax one set of muscles, and leave others comparatively idle. The very best course to pursue is, to take a part in every thing going on, in fact, "every thing by turns, but nothing long." One of the most important items of advice that can be given in this connection is, that an hour or two should be spent in walking in the open air, at two or three different times, until the very last. Nothing so certainly, so safely, and so pleasantly contributes to an easy deliverance. A volume could be given of the most strikingly illustrative facts; but the single sentence must suffice. Let it be pondered well; let the father insist upon it, encourage it, and do all he can to make its performance easy and agreeable. These, with regular, daily, bodily habits, would add incalculably to the sum total of human happiness; whilst, by their neglect, by simply pass ing the time in eating, lounging, and listlessness, in the wearing, irritating inactivities of a boarding-house, or hotel life, monsters in bodily shape, and imbeciles in mind, are constantly thrown out on society, to be disgusted by their presence, or to be taxed by their confinement in some insane retreat, or some friendly asylum.

As certainly as one end of a telegraph-wire is answered at the other, so certainly do the nervous conditions of the new being and the parent answer to one another, only with this difference: the telegraph responds from either end; in the case in hand, influences go out from the parent only. What kind of a character, then, shall be impressed on the coming man depends upon the abiding states of mind of the mother. The material was made to her hand; it is her part to mold it; to her are the destinies of this coming man committed, and the responsibilities are fearful. She gives the hues to an existence which is immortal and which it must bear for good or ill all along the way of that immortality, saving the modifications which Divinity may make. The true mother, then, will not at such an interesting, such a momentous period of her existence, allow her mind to be absorbed in questions of what she shall eat and what she shall drink, and thus give a gourmand to the world; she will not luxuriate in the frivolities of dress, in the study of the fashions, the dissipations of society, nor yield herself to the seductions of the courtier, the flatterer, and the ladies' man, and thus add another to the throng of the giddy-minded, the empty-headed, and the inane; nor let her not pine in pettishness and anger for what is now beyond her reach; for a position in circles and sets above her present sphere; let her not call in question the wisdom, the benevolence, or the justice of the wise and kind Father of all for her allotment in life; let her not employ the mind in irritating and wearing envies and jealousies, in carping criticisms, in wearing, wasting complaints, in oppressive forebodings of ills to come; let her, on the contrary, war against all these with the whole energy of her nature, regarding them as her worst enemies and the bane of domestic life. Let her constantly look at the sunshine and the sky, the leaf and the flower; let her take the first step toward all true elevation, the contemplation of individual unworthiness of any blessing the merciful One could bestow; then look around upon the innumerable ones enjoyed; and next wake up in gladsome gratitude, that such a profusion of goodness should come to one so insignificant, from the generous hand of Omnipotence. Then there will begin to flow in upon the heart all the time, a perfect flood of elevating emotions; there will be joy and gladness; there will be life and light; there will be mirth and song; there will be mercy and magnanimity; there will be sympathy and beneficence; and purity and truth, generosity and nobleness of nature will color the whole character, to be perpetuated in a long line of generations to come. A mother's responsibility! who can measure it? She has the molding of the race, for good or ill, in a measure only second to the God who made her! And honored far, far above kings and conquerers and potentates be she, however lowly may be her position among the millions of earth, who most deeply feels these responsibilities, and who most humbly endeavors to perform them according to her ability, leaning, meanwhile and always on Him, whose kingdom ruleth over all.

